# Dynamic changes of cytokine profiles and virological markers during 48 weeks of entecavir treatment for HBeAg-positive chronic hepatitis B

**DOI:** 10.3389/fimmu.2022.1024333

**Published:** 2022-09-20

**Authors:** Minghui Li, Yuanjiao Gao, Liu Yang, Yanjie Lin, Wen Deng, Tingting Jiang, Xiaoyue Bi, Yao Lu, Lu Zhang, Ge Shen, Ruyu Liu, Shuling Wu, Min Chang, Mengjiao Xu, Leiping Hu, Rui Song, Yuyong Jiang, Wei Yi, Yao Xie

**Affiliations:** ^1^ Department of Hepatology Division 2, Beijing Ditan Hospital, Capital Medical University, Beijing, China; ^2^ Department of Hepatology Division 2, Peking University Ditan Teaching Hospital, Beijing, China; ^3^ Department of Infectious Disease, Beijing Ditan Hospital, Capital Medical University, Beijing, China; ^4^ Center of Integrative Medicine, Beijing Ditan Hospital, Capital Medical University, Beijing, China; ^5^ Department of Gynecology and Obstetrics, Beijing Ditan Hospital, Capital Medical University, Beijing, China

**Keywords:** chronic hepatitis B, cytokines, entecavir, hepatitis B e antigen, deoxyribose nucleic acid

## Abstract

**Objective:**

The aims of this study were to investigate the kinetic changes of serum, virological, and immunological markers during entecavir (ETV) antiviral therapy and to explore whether these indicators can predict the antiviral efficacy of ETV in hepatitis B e antigen (HBeAg)-positive chronic hepatitis B (CHB) patients.

**Methods:**

HBeAg-positive CHB patients were enrolled and treated with ETV 0.5 mg/day. Clinical biochemical, virological, and serological tests were performed at baseline and every 12 weeks during the 48-week treatment. Plasma levels of cytokines (Flt-3L, IFN-α2, IFN-γ, IL-10, IL-17A, IL-6, TGF-β1, TGF-β2, TGF-β3, and TNF-α) were measured at baseline and at 12 and 24 weeks after treatment. Analysis of the trends of these clinical indicators in ETV antiviral therapy was performed.

**Results:**

A total of 105 HBeAg-positive CHB patients were enrolled, and 100 of them completed 48 weeks of ETV treatment and follow-up. After 48 weeks of treatment, hepatitis B s antigen (HBsAg) decline ≥ 1 log10 was found in seven patients, but no patient achieved HBsAg disappearance. serological HBeAg disappeared in 13 patients, and serological HBeAg transformed in 3 patients. The baseline HBsAg and HBeAg levels, HBV DNA load, IL-10, and TGF-β1 levels in the complete virological response group were lower than those in the incomplete virological response group, while the ALT level in the complete virological response group was higher than that in the incomplete virological response group. Both univariate analysis and multivariate analysis showed that baseline biochemical indexes, virological indexes, and cytokine levels had no correlation with the complete virological response at 48 weeks. In multivariate analysis, low baseline HBV DNA load, and HBeAg and IL-10 levels were significantly associated with ALT normalization after 48 weeks of ETV treatment (HBeAg OR = 1.003, 95% CI 1.001–1.006, *p* = 0.007; HBV DNA OR = 0.184, 95% CI 0.046–0.739, *p* = 0.017; IL-10 OR = 0.040, 95% CI 0.972–0.999, *p* = 0.040).

**Conclusion:**

Cytokine levels changed dynamically during ETV antiviral therapy. Low baseline HBV DNA load, and HBeAg and IL-10 levels were significantly associated with ALT normalization after 48 weeks of ETV treatment.

## Introduction

Chronic hepatitis B (CHB) is one of the most serious infectious diseases in China (1, 2). CHB is considered to be an immune-mediated disease, in which the immune response initiated by HBV causes hepatocyte necrosis and inflammation of liver tissue. It causes liver tissue damage while clearing the virus. If there is no immune clearance, long-term HBV infection will not cause liver histological damage (3–5).

Antiviral therapy can significantly reduce the incidence of liver cirrhosis and liver cancer in patients with CHB, and is an important means to delay the progression of liver disease. Interferon and nucleoside (acid) analogs are two types of effective antiviral drugs for CHB as recommended by most guidelines (2, 6, 7). We previously found that the pathogenesis and antiviral efficacy of CHB were related to immune cells and cytokines. The pathogenesis of CHB was positively correlated with interferon-alpha (IFN-α) and negatively correlated with transforming growth factor beta (TGF-β) and interleukin-10 (IL-10) (8, 9), while TGF-β and interferon-gamma (IFN-γ) were related to the efficacy of interferon (10). Our study also found that, compared with entecavir (ETV) therapy, peginterferon-alpha (PEG-IFN-α) treatment could significantly increase natural killer (NK) cell frequency and function (11). Recently, we have reported that patients with lower baseline hepatitis B virus deoxyribose nucleic acid (HBV DNA) load and higher baseline cluster of differentiation86+ (CD86+) plasmacytoid dendritic cell (pDC) % were more likely to obtain functional cure (12), and the dynamic changes of early cytokines and virological markers were associated with clinical cure of hepatitis B e antigen (HBeAg)-positive CHB (13). These results indicate that pathogenesis of CHB and antiviral treatment especially the interferon treatment is related to immunity.

It remains to be determined whether the effect of nucleoside (acid) analogues, another class of first-line drugs for antiviral treatment of CHB, is related to immunity. The purpose of this study is to explore the changes in serology, virology, and cytokine indexes in patients with HBeAg-positive CHB during the 48-week ETV treatment. We detected cytokines that cause liver inflammation and immunosuppression (IL-6, IL-10, TNF-α, and TGF-β), factors that stimulate immune cell function (IFN-α), and factors that have viral clearance (IL-17A and IFN-γ) or can stimulate proliferation of DC cells and NK cells (Flt-3L) (14–17). We hope to find out the serology, virology, and cytokine indexes related to ETV antiviral response, and to provide a basis for the selection of a more reasonable antiviral treatment scheme.

## Materials and methods

### Subjects and inclusion and exclusion criteria

Subjects of this prospective cohort study were HBeAg-positive patients with CHB. From November 2017 to November 2018, HBeAg-positive CHB patients who were willing to be treated with ETV in the Department of Hepatology Division 2 of Beijing Ditan Hospital Affiliated to Capital Medical University entered the study after signing informed consent.

The inclusion criteria of HBeAg-positive CHB patients were as follows (15): (1) continuous hepatitis B s antigen (HBsAg) positive (HBsAg ≥ 0.05 IU/ml) > 6M; (2) HBeAg positive (HBeAg ≥ 1.0 S/CO); (3) HBV DNA positive (>10^4^ IU/ml); (4) ALT abnormality (≥80 IU/L) lasting for more than 3 months or significant inflammation (above G2) in liver histological examination; (5) 18–65 years old; (6) gender is not limited; and (7) no use of hormones and/or immunosuppressants and other liver protection drugs.

Exclusion criteria were as follows: (1) complicated with other virus (EBV, CMV, HIV, HCV, HDV, etc.) infection; (2) combined with autoimmune liver disease; (3) chronic alcoholism; (4) taking other liver damaging drugs; (5) have a history of mental illness; (6) have evidence of liver tumor (clinical diagnosis of liver cancer or AFP > 100 ng/ml); (7) liver fibrosis and cirrhosis (excluded by Fibroscan) (18); (8) those who have serious heart, brain, lung, kidney, and other serious diseases and cannot participate in long-term follow-up; (9) use of hormones and/or immunosuppressants; and (10) there are other liver diseases (fatty liver, metabolic liver disease, and hereditary liver disease).

This study was approved by the Ethics Committee of Beijing Ditan Hospital affiliated to Capital University of Medical Sciences (JDL-2017-034-01), and was registered with Clinical Trials (NCT03210506).

### Research design

The patients were treated with ETV 0.5 mg/day and followed up for 48 weeks after enrollment. The virological and serological indexes, biochemistry, and AFP were tested at baseline and every 3 months during ETV treatment. Liver imaging examination was performed every 6 months. Cytokines (Flt-3L, IFN-α2, IFN-γ, IL-10, IL-17A, IL-6, TGF-β1, TGF-β2, TGF-β3, and TNF-α) were measured at baseline and at the 3rd and 6th months of treatment to analyze the change trend of cytokines in ETV antiviral therapy.

Grouping: According to HBV DNA level at 48 weeks of ETV treatment, the patients were divided into complete virological response group with HBV DNA < 20 IU/ml and incomplete virological response group with HBV DNA ≥ 20 IU/ml. According to the ALT level at 48 weeks of ETV treatment, the subgroups were divided into normal ALT group with ALT < 40 U/L and abnormal ALT group with ALT ≥ 40 U/L.

This study mainly observed the response of patients during 48 weeks of treatment. After 48 weeks of treatment, the ETV treatment would continue if the response was good. If the response was poor, ETV would be switched to other nucleosides (acids) or interferon alone/in combination according to the patient’s wishes.

This is a prospective exploratory study. We previously found that, approximately 80% of CHB patients achieved virological response (clinical outcome event) after 48 weeks of ETV treatment. Six factors (age, gender, HBsAg, HBeAg, HBV DNA, and immune cells) affecting the effect of ETV on CHB treatment were considered, and 15 patients were enrolled for each factor (15 × 6 = 90 patients). Considering a loss rate of 10%–20%, the sample size was determined to be 99–118 cases. In the end, we enrolled 105 patients with CHB in this prospective study, which met the statistical requirements.

### Detection of HBV DNA, HBV serology, and clinical indexes

Blood routine (Sysmex Corporation, Japan), liver function (Wako Pure Chemical Industries, Ltd., Japan), renal function (Sekisui Medicalcal Co, Ltd, Japan), tumor series index (Abbott Ireland Diagnostics Division, Finisklin Business Park, Sligo, Ireland), and serum HBV DNA load (Cobas ampliprep/Cobas TaqMan 96, polymerase chain reaction, PCR, detection limit < 20 IU/ml) were performed.

HBsAg/anti HBs level and HBeAg/anti-HBe level were detected by Abbott Architac i2000 microparticle chemiluminescence reagent. The detection range of HBsAg level is 0.05–250 IU/ml; those with HBsAg level greater than 250 IU/ml will be automatically diluted 500 times. The actual HBsAg level is calculated by multiplying the detection value by 500. HBsAg < 0.05 IU/ml was defined as the disappearance of HBsAg.

### Quantitative detection of plasma cytokines

The following cytokines were detected by Luminex Technology: Fms-like tyrosine kinase 3 ligand (Flt-3L), interferon-alpha 2 (IFN-α2), interferon-gamma (IFN-γ), interleukin-10 (IL-10), interleukin-17A (IL-17A), interleukin-6 (IL-6), tumor necrosis factor alpha (TNF-α), transforming growth factor beta1 (TGF-β1), transforming growth factor beta2 (TGF-β2), and transforming growth factor beta 3 (TGF-β3). All the data were analyzed by Flexmap 3D analyzer.

Our study aimed to investigate whether cytokines can early predict the efficacy of ETV therapy for HBeAg-positive chronic hepatitis B; thus, we tested cytokines primarily at baseline, week 12, and week 24.

### Statistical analysis

The counting data were expressed by the number of cases and the percentage, and the Chi-square test was used for comparison between groups. The statistical description of normal distribution measurement data was expressed by means ± standard deviation (mean ± SD), and the *t*-test of two independent samples was used for comparison between the two groups. Non-normal distribution data were expressed by median and quartile (median, Q1Q3), and Mann–Whitney *U* test was used for comparison between groups. Spearman or Pearson correlation test was used for the correlation of variables according to whether they conformed to normal distribution.

All data were statistically analyzed with SPSS (Statistical Package for the Social Sciences) 21.0 software (SPSS, Chicago, IL) and GraphPad Prism 5 software. In the statistical analysis of the data, all tests were performed by two-sided test.

Bonferroni is used to correct the rank-sum test results at each different time point, and the correction level is *α*. Among them, biochemical, viral, serological, and other indicators were observed at five time points. *α* was set to 0.01, and *p* < 0.01 was statistically significant. There were three observation time points for cytokines. *α* was set as 0.013, and *p* < 0.013 was statistically significant.

## Results

### Basic clinical characteristics of patients

**Figure 1:**
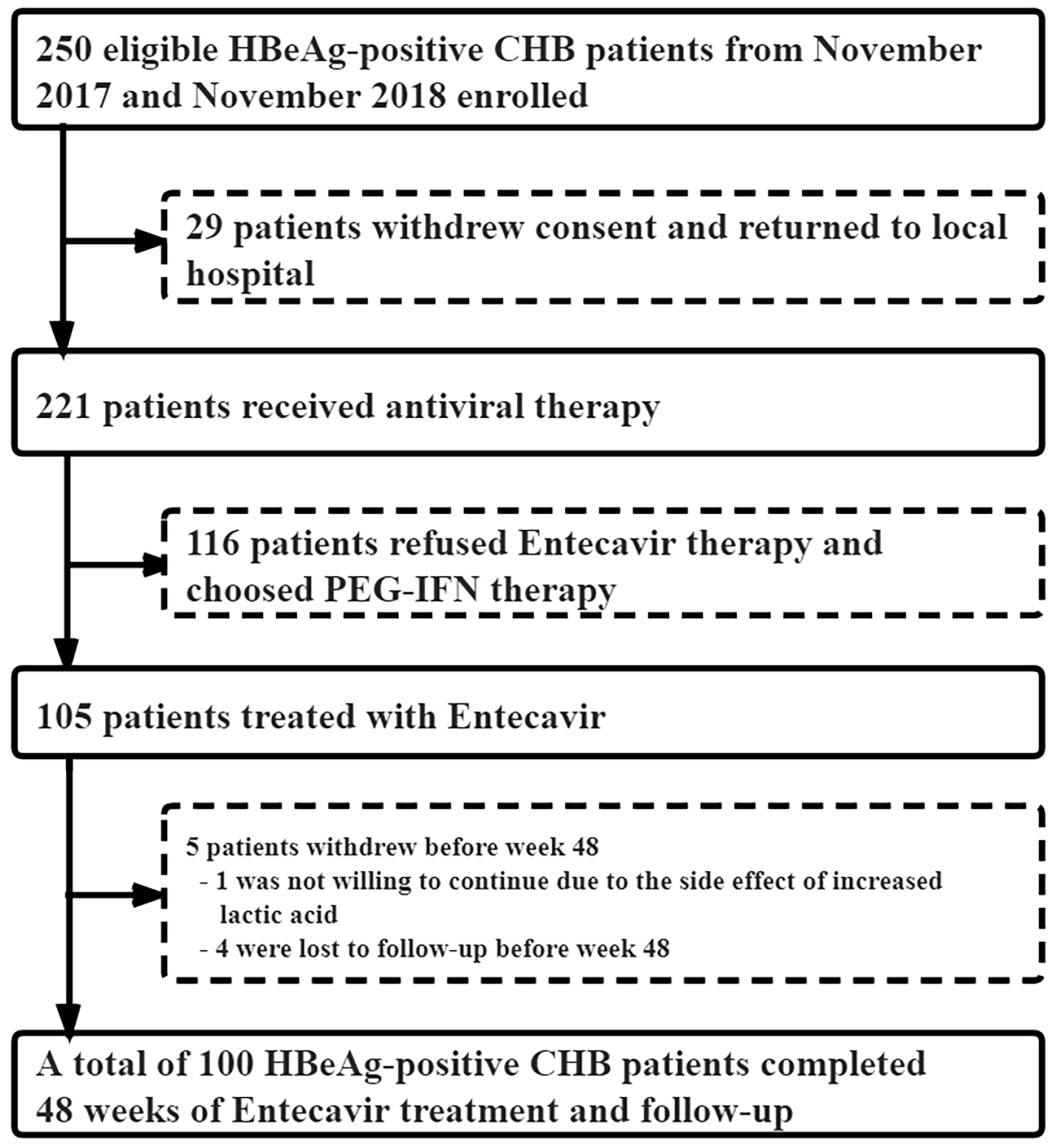
Patient enrollment and deposition.

From November 2017 to November 2018, 221 of the 250 eligible HBeAg-positive CHB patients signed the informed consent to enter our study, and the remaining 29 were lost to follow-up. Among the 221 CHB patients, 105 received ETV antiviral therapy, while 116 chose to be treated with PEG-IFNα-2a. In the ETV group, one case withdrew due to the increase in lactate, and four cases did not complete the 48-week follow-up. Finally, a total of 100 CHB patients treated with ETV completed the 48-week follow-up (**Figure 1**). The median age was 32 years (28–38), with 61 men and 39 women. After 48 weeks of treatment, HBsAg decreased by ≥1 log10 in seven cases, but no patient achieved HBsAg disappearance. serological HBeAg disappeared in 13 cases, and serological HBeAg transformed in 3 cases.

### Group analysis according to virological response at week 48 of ETV treatment

**Figure 2:**
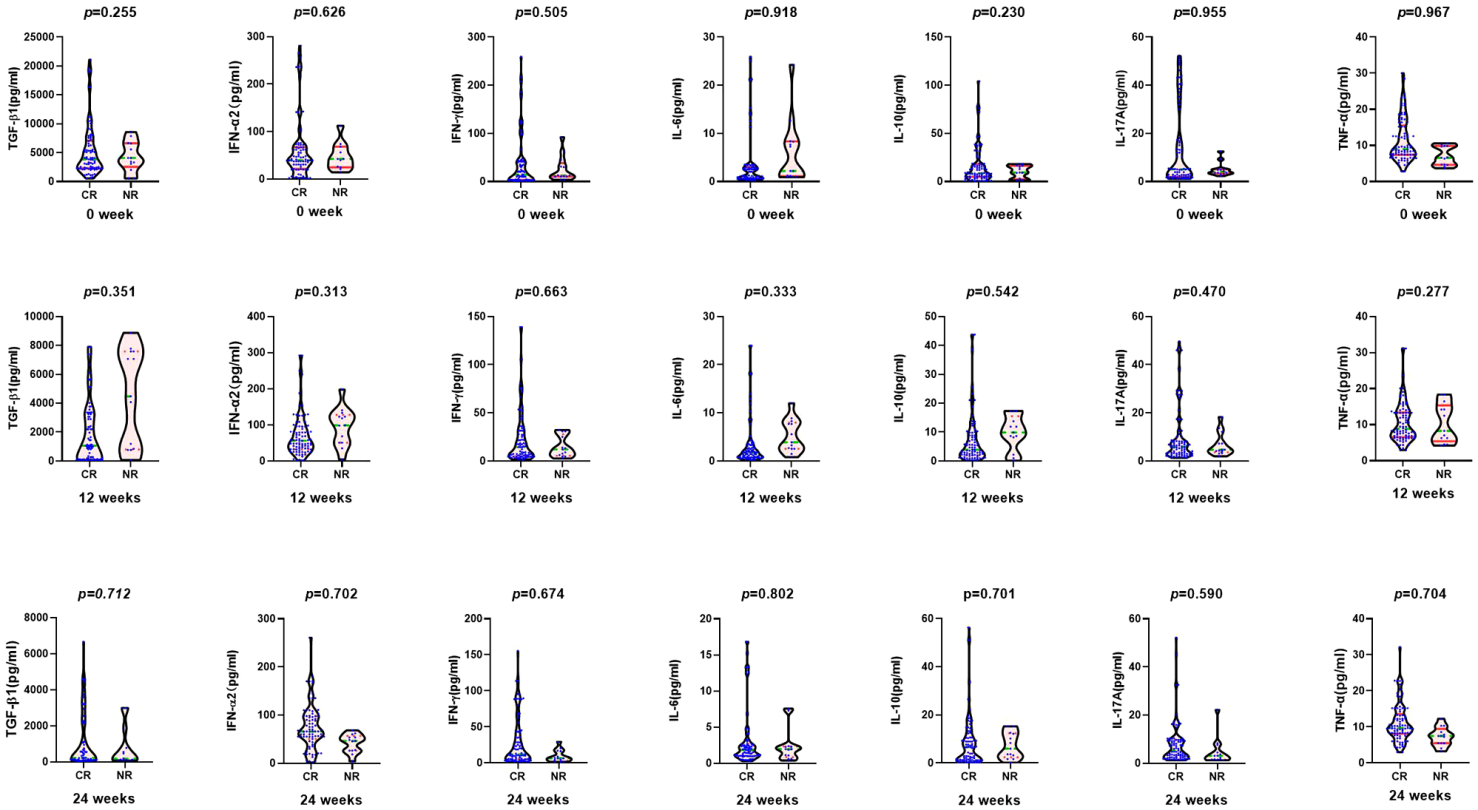
Comparison of cytokines between complete virological response group (CR: *n* = 87) and non-complete virological response group (NR: *n* = 13) at baseline, 12, and 24 weeks after entecavir therapy.

According to the HBV DNA level at 48 weeks of ETV treatment, the complete virological response group was defined as HBV DNA < 20 IU/ml, and the incomplete virological response group was defined as HBV DNA ≥ 20 IU/ml. There were 87 cases (63 men) in the complete virological response group and 13 patients (8 men) in the incomplete virological response group. There were no statistical differences in age, biochemical indexes (ALT, AST, TBIL, and ALB), virological indexes (HBV DNA, HBsAg, and HBeAg), or cytokines between the two groups at baseline and 3 months (**Figure 2**).

At the 6th month, there was significant difference in the median HBeAg between the two groups (57.67 *vs*. 291.05 S/CO, *Z* = −2.260, *p* = 0.024), but not in HBsAg level, biochemical indexes, or cytokines (**Figure 2**). After 9 months, the median HBeAg and HBsAg levels in the complete virological response group were significantly lower than those in the incomplete virological response group (HBeAg 22.67 *vs*. 886.21 S/CO, *Z* = −4.895, *p* = 0.000; HBsAg 3.66 log10 *vs*. 4.06 log10 IU/ml, *Z* = −4.034, *p* = 0.017).

After 12 months of treatment, the median HBeAg and HBsAg levels were significantly lower in the complete virological response group as compared with the incomplete virological response group (HBeAg 11.25 *vs*. 906.46 S/CO, *Z* = −5.007, *p* = 0.000; HBsAg 3.64 log10 *vs*. 4.09 log10 IU/ml, *Z* = −3.645, *p* = 0.000), and the median AST was significantly increased (21 *vs*. 17.40 U/L, *Z* = −2.266, *p* = 0.023). However, there was no difference in ALT, TBil, and ALB between the two groups (**Table 1**).

**Table 1:** Comparison of clinical indicators between the complete virological response group (*n* = 87) and incomplete virological response group (*n* = 13) at baseline and 12, 24, 36, and 48 weeks after entecavir therapy.

	Baseline			12 weeks			24 weeks			36 weeks			48 weeks		
	Complete virological response	Incomplete virological response	Z/*p*	Complete virological response	Incomplete virological response	Z/*p*	Complete virological response	Incomplete virological response	Z/*p*	Complete virological response	Incomplete virological response	Z/*p*	Complete virological response	Incomplete virological response	Z/*p*
**ALT (U/L)**	229.30 (121.20–360.94)	217 (153.55–324)	–0.164/0.870	32 (21–49.8)	43.2 (27.35–80.75)	−1.604/0.109	32 (21–49.8)	43.2 (27.35–80.75)	−1.082/0.279	22.40 (15.80–28.90)	24.90 (22.40–30.80)	−1.528/0.127	20.50 (15.30–29.10)	23.10 (16.90–25.60)	0.000/1.000
**AST (U/L)**	101.75 (61.18–195.63)	112.70 (50.35–137.50)	–0.815/0.415	27.7 (19.90–35.50)	30 (21.95–38.75)	−0.856/0.392	27.7 (19.90–35.50)	30 (21.95–38.75)	−1.374/0.169	21 (16.90–25.40)	22.40 (15.15–25.20)	−0.220/0.826	21.40 (17.60–25.00)	17.40 (16.45–20.70)	−2.266/0.023
**TBil (µmol/L)**	15.70 (11.55–21.43)	13.70 (11.60–22.85)	–0.164/0.870	12.6 (9.3–18.20)	12.8 (8.40–23.15)	−0.385/0.701	12.6 (9.3–18.20)	12.8 (8.40–23.15)	−1.374/0.169	13 (10.00–16.20)	12.5 (8.95–13.50)	−1.133/0.257	12.20 (9.90–14.90)	13.50 (9.95–17.50)	−0.723/0.470
**ALB (g/L)**	45.80 (40.98–48.40)	45.00 (43.05–46.95)	0.218/0.828	47.8 (44.50–49.20)	45.6 (43.65–48.25)	−1.539/0.124	47.8 (44.50–49.20)	45.6 (43.65–48.25)	−1.539/0.124	48.50 (46–50.20)	48.50 (46.50–49.85)	−0.062/0.951	48.20 (46.40–49.80)	49.70 (47.40–49.90)	−1.661/0.097
**HBsAg (log10 IU/ml)**	3.86 (3.59–4.10)	3.94 (3.70–4.10)	–0.677/0.499	3.77 (3.30–4.04)	3.65 (3.52–3.93)	−0.113/0.910	3.77 (3.30–4.04)	3.65 (3.52–3.93)	−0.379/0.704	3.66 (3.22–3.92)	4.06 (3.99–4.37)	−4.034/0.000	3.64 (3.18–3.92)	4.09 (3.80–4.27)	−3.645/0.000
**HBeAg (S/CO)**	811.69 (437.33–1,173.23)	805.87 (683.66–1,148.33)	–0.502/0.615	57.67 (13.74–665.09)	291.05 (50.02–931.78)	−1.596/0.111	57.67 (13.74–665.09)	291.05 (50.02–931.78)	−2.260/0.024	22.67 (3.40–96.65)	886.21 (321.53–1,190.18)	−4.895/0.000	11.25 (2.30–50.24)	906.46 (164.32–1,194.36)	−5.007/0.000
**HBV DNA (log10 IU/ml)**	6.63 (6.34–6.96)	7.99 (7.12–8.36)	–3.536/<0.001	2.75 (0–3.34)	4.29 (3.94–4.59)	−4.344/<0.001	0.00 (0.00,2.36)	3.72 (2.77–3.79)	−4.639/<0.001	0.00 (0.00, 0.00)	3.32 (2.77–3.79)	−7.192/<0.001	0.00 (0.00, 0.00)	3.07 (2.49–3.44)	−9.918/<0.001
**Flt-3L (pg/ml)**	0.11 (0.02–43.59)	NA	NA	1.25 (0.02–28.46)	NA	−1.225/0.333	2.24 (0.02–78.56)	NA	−1.225/0.221	/	/	/	/	/	/
**IFN-α2 (pg/ml)**	41.60 (26.46–74.19)	62.21 (26.21–74.21)	–0.487/0.626	56.01 (34.02–98.29)	72.34 (40.88–112.53)	−1.010/0.313	67.89 (52.61–98.74)	46.38 (26.46–56.88)	−0.382/0.702	/	/	/	/	/	/
**IFN-γ (pg/ml)**	19.72 (3.92–45.38)	30.05 (5.89–161.61)	–0.666/0.505	14.43 (5.79–31.55)	10.01 (4.59–24.97)	−0.436/0.663	12.94 (5.22–45.28)	6.47 (3.25–16.70)	−0.420/0.674	/	/	/	/	/	/
**IL-10 (pg/ml)**	8.58 (4.94–17.69)	15.60 (8.53–21.89)	–1.199/0.230	4.70 (2.08–10.28)	6.12 (2.54–13.31)	−0.610/0.542	6.56 (1.23–12.56)	5.98 (2.40–12.37)	−0.384/0.701	/	/	/	/	/	/
**IL-17A (pg/ml)**	5.20 (2.73–37.25)	5.20 (2.60–25.94)	–0.056/0.955	6.10 (3.12–10.12)	4.09 (2.86–10.47)	−0.723/0.470	6.28 (2.86–10.13)	2.29 (1.54–4.05)	−0.538/0.590	/	/	/	/	/	/
**IL-6 (pg/ml)**	2.44 (0.84–7.25)	2.55 (0.71–5.59)	–0.103/0.918	1.89 (0.80–3.76)	2.20 (1.40–7.08)	−0.969/0.333	1.92 (1.02–3.20)	1.85 (0.84–2.32)	−0.251/0.802	/	/	/	/	/	/
**TNF-α (pg/ml)**	8.79 (6.59–13.30)	8.64 (6.86–15.81)	–0.041/0.967	8.29 (6.29–13.67)	12.51 (6.93–15.95)	−1.087/0.277	9.72 (7.69–14.24)	7.53 (5.48–10.30)	−0.379/0.704	/	/	/	/	/	/
**TGF-β1 (pg/ml)**	3,874 (2,314–7,019)	5,025 (2,786–10,500)	–1.138/0.255	1,455 (203.13–3,767)	889 (105.47–3,401.50)	−0.933/0.351	468 (103.86–2,548)	170.12 (104.78–1,328.32)	−0.369/0.712	/	/	/	/	/	/
**TGF-β2 (pg/ml)**	412.21 (217.39–575.38)	NA	NA	1.25 (0.02–28.46)	NA	−1.225/0.333	/	/	/	/	/	/	/	/	/
**TGF-β3 (pg/ml)**	187.12 (151.96–209.12)	NA	NA	56.01 (34.02–98.29)	72.34 (40.88–112.53)	−1.010/0.313	/	/	/	/	/	/	/	/	/

Biochemistry and virus indicators: p < 0.010 was statistically significant. Cytokines: p < 0.013 was statistically significant.

### Baseline predictors of complete virological response at week 48 of ETV treatment

Both univariate analysis and multivariate analysis showed that baseline biochemical indexes (ALT, AST, TBIL, and ALB), virological indexes (HBV DNA, HBsAg, and HBeAg), and cytokine levels had no correlation with the complete virological response after 48 weeks (**Table 2**).

**Table 2:** Logistic regression analysis of the baseline predictors of complete virological response after 48 weeks of ETV treatment.

Univariate analysis	OR	95% CI	*p*-value
**HBsAg (log10 IU/ml)**	1.993	0.437–9.092	0.373
**HBeAg (S/CO)**	1.001	0.999–1.002	0.417
**HBV DNA (log10 IU/ml)**	1.550	0.766–3.136	0.223
**ALT (U/L)**	0.999	0.995–1.002	0.385
**AST (U/L)**	0.996	0.990–1.003	0.251
**TBil (µmol/L)**	0.994	0.924–1.068	0.862
**ALB (g/L)**	1.014	0.894–1.150	0.829
**Flt-3L (pg/ml)**	1.013	0.948–1.083	0.704
**IFN-α2 (pg/ml)**	0.997	0.991–1.003	0.374
**IFN-γ (pg/ml)**	1.000	0.997–1.004	0.849
**IL-10 (pg/ml)**	1.000	0.985–1.015	0.993
**IL-17A (pg/ml)**	0.995	0.981–1.009	0.499
**IL-6 (pg/ml)**	0.996	0.980–1.011	0.586
**TNF-α (pg/ml)**	0.988	0.919–1.062	0.742
**TGF-β1 (pg/ml)**	1.000	1.000–1.000	0.297
**TGF-β2 (pg/ml)**	1.004	0.991–1.018	0.539
**TGF-β3 (pg/ml)**	0.991	0.919–1.068	0.805
**Multiple factor analysis**	OR	95% CI	*p*-value
**HBV DNA (log10 IU/ml)**	1.342	0.592–3.042	0.481

ALT, glutamic–pyruvic transaminase; AST, glutamic oxalacetic transaminase; TBil, total bilirubin. ALB, albumin.

### Subgroup analysis of ALT level at week 48 of ETV treatment

The normal ALT values for men and women in different regional guidelines are inconsistent. The 2016 AASLD guidelines suggest that the upper limit of normal ALT values for men and women with HBV infection is 30 U/L and 19 U/L, respectively (19). The 2018 AASLD guidelines suggest that the normal ALT values for infected men and women are 35 U/L and 25 U/L (7), respectively. However, the Asia-Pacific guidelines and EASL guidelines have always used 40 U/L as the normal upper limit of ALT levels for men and women (6, 20). Since ALT levels are the most sensitive indicator of hepatocyte necrosis and liver inflammation, patients with abnormal ALT and more pronounced ALT elevations often have more significant liver inflammation than patients with normal ALT level and mildly elevated ALT. Therefore, as proposed in the 2015 APASL and 2017 EASL guidelines, 40 U/L was still used as the normal upper limit of ALT level in this study.

According to the ALT level at 48 weeks of ETV treatment, the subgroups were divided into the normal ALT group with ALT < 40 U/L and the abnormal ALT group with ALT ≥ 40 U/L. There were 90 patients (64 men) in the normal ALT group and 10 patients (7 men) in the abnormal ALT group. They had similar age, biochemical indexes, and cytokines at baseline. The baseline HBsAg and HBeAg levels in the ALT normalization group were significantly lower than that in the non-normalization group. After 3 months, the median HBsAg and HBeAg levels in the normal ALT group were significantly higher as compared with the abnormal ALT group (HBsAg 3.78 log10 *vs*. 3.29 log10 IU/ml, *Z*= −2.632, *p* = 0.008; HBeAg 86.66 *vs*. 12.85 S/CO, *Z* = −2.654, *p* = 0.008), and the median TBIL level was significantly lower (TBiL 12.20 *vs*. 25.50 µmol/L, *Z* = −2.477, *p* = 0.013).

After 6 months, the median HBsAg and HBeAg levels in the normal ALT group were significantly higher than those in the abnormal ALT group (HBsAg 3.84 log10 *vs*. 3.23 log10 IU/ml, *Z* = −2.356, *p* = 0.018; HBeAg 86.74 *vs*. 12.56 S/CO, *Z* = −2.287, *p* = 0.022).

After 9 months, the median HBeAg level in the normal ALT group was significantly higher compared with the abnormal ALT group (57.13 *vs*. 8.76 S/CO, *Z* = −1.976, *p* = 0.048).

At 12 months, the median HBsAg level in the normal ALT group was significantly higher as compared with the abnormal ALT group (3.68 log10 *vs*. 3.04 log10 IU/ml, *Z* = −2.195, *p* = 0.028), while the median ALT and AST levels were lower (ALT 20.10 *vs*. 48.20 U/L, *Z* = −5.172, *p* = 0.000; AST 20.70 *vs*. 41.90 U/L, *Z* = −4.862, *p* = 0.000) (**Table 3**).

**Table 3:** Comparison of clinical indicators between the normal ALT group (*n* = 90) and abnormal ALT group (*n* = 10) at baseline and weeks 12, 24, 36, and 48 during entecavir therapy.

	Baseline			12 weeks			24 weeks				36 weeks			48 weeks		
	Normal ALT	Abnormal ALT	*Z*/*p*	Normal ALT	Abnormal ALT	*Z*/*p*	Normal ALT	Abnormal ALT		*Z*/*p*	Normal ALT	Abnormal ALT	*Z*/*p*	Normal ALT	Abnormal ALT	*Z*/*p*
**ALT (U/L)**	300.55 (201–743.29)	225.60 (117.65–345.20)	−1.827/ 0.068	31.90 (22.70–49.90)	41.90 (15.50–86.15)	−0.644/ 0.520	23.05 (17.85–34.05)	22.40 (11.00–45.50)		−0.483/ 0.629	22.75 (16.90–28.90)	24.30 (13.10–35.40)	−0.172/ 0.863	20.10 (15.10–26.45)	48.20 (42.45–75.40)	−5.172/ 0.000
**AST (U/L)**	126.60 (88.20–625.80)	100.50 (54.20–179.20)	−1.623/ 0.103	27.70 (19.93–35.38)	29.70 (19.10–43.10)	−0.689/ 0.491	22.45 (17.90–27.30)	23.65 (16.30–34.10)		−0.517/ 0.605	20.90 (16.38–25.60)	21.00 (16.90–25.40)	−0.477/0.633	20.70 (16.50–22.88)	41.90 (29.13–46.80)	−4.862/ 0.000
**TBil (µmol/L)**	21.20 (18.90–28.28)	14.30 (11.40–21.05)	−2.534/ 0.011	12.20 (9.10–17.85)	25.50 (9.30–32.78)	−2.477/ 0.013	12.20 (9.50–15.50)	20.90 (8.80–26.68)		−1.644/ 0.100	12.80 (10.30–15.50)	15.50 (7.30–26.98)	0.425/ 0.671	12.20 (9.90–14.80)	19.45 (7.90–22.05)	−1.604/ 0.109
**ALB (g/L)**	41.25 (39.38–48.93)	45.90 (42.85–47.85)	−1.446/0.148	47.80 (44.30–49.10)	44.70 (44.50–49.93)	−0.290/ 0.772	47.75 (45.60–49.03)	47.50 (45.30–50.53)		−0.397/ 0.692	48.50 (46.40–50.20)	48.45 (43.60–49.40)	−0.948/0.343	48.50 (46.50–49.83)	47.85 (45.90–49.80)	−0.523/ 0.601
**HBsAg (log10 IU/ml)**	3.76 (2.87–3.91)	3.89 (3.65–4.10)	−2.000/ 0.045	3.78 (3.28–4.04)	3.29 (3.08–3.68)	−2.632/ 0.008	3.84 (3.39–4.06)	3.23 (3.02–3.85)		−2.356/ 0.018	3.77 (3.45–4.01)	3.16 (3.11–3.92)	−1.937/0.053	3.68 (3.37–3.96)	3.04 (3.02–3.86)	−2.195/ 0.028
**HBeAg (S/CO)**	358.26 (322.81–651.35)	908.07 (589.15–1,222.08)	−2.896/ 0.004	86.66 (17.01–719.60)	12.85 (0.78–133.77)	−2.654/ 0.008	86.74 (7.09–462.31)	12.56 (1.00–98.95)		−2.287/ 0.022	57.13 (4.36–233.87)	8.76 (1.27–78.81)	−1.976/ 0.048	22.25 (4.49–136.72)	9.09 (1.11–30.01)	−1.839/ 0.066
**HBV DNA (log10 IU/ml)**	6.65 (6.33–7.32)	6.70 (6.45–7.51)	−0.460/0.646	3.01 (1.12–3.77)	2.76 (2.38–3.06)	−0.468/0.640	1.54 (0.00–2.69)	2.36 (0.00–2.68)		−0.290/0.772	0.00 (0.00–1.65)	0.00 (0.00–0.58)	−0.647/0.518	0	0	−1.278/0.201
**Flt-3L (pg/ml)**	0.07 (0.02–41.09)	NA	NA	0.64 (0.02–1.25)	NA	NA	1.13 (0.02–2.24)	NA		NA	/	/	/	/	/	/
**IFN-α2 (pg/ml)**	45.79 (29.69–76.08)	30.72 (4.95–62.21)	−2.029/ 0.043	57.85 (33.60–98.39)	47.90 (33.37–86.51)	−0.230/ 0.818	58.34 (36.00–94.46)	71.69 (60.13–119.50)		−1.708/ 0.088	/	/	/	/	/	/
**IFN-γ (pg/ml)**	20.16 (3.92–51.00)	20.23 (2.39–107.04)	−0.402/ 0.688	14.43 (6.12–31.55)	4.71 (3.23–47.09)	−1.735/ 0.083	14.43 (4.79–43.00)	10.01 (5.34–50.19)		−0.494/ 0.621	/	/	/	/	/	/
**IL-10 (pg/ml)**	9.07 (4.94–17.69)	13.25 (1.01–121.22)	−0.322/ 0.748	5.47 (2.16–10.28)	4.93 (0.92–81.82)	−0.149/ 0.881	5.30 (1.60–12.30)	6.22 (1.12–49.62)		−0.891/0.373	/	/	/	/	/	/
**IL-17A (pg/ml)**	5.18 (2.75–35.74)	14.68 (1.25–39.77)	−0.144/ 0.886	6.47 (3.22–10.12)	4.09 (1.31–15.51)	−1.385/ 0.166	6.15 (2.56–9.71)	5.09 (2.12–11.70)		−0.408/0.683	/	/	/	/	/	/
**IL-6 (pg/ml)**	2.49 (0.90–7.83)	1.46 (0.62–2.68)	−1.149/ 0.250	1.90 (0.88–3.79)	1.40 (0.58–11.75)	−0.345/ 0.730	2.01 (1.02–2.69)	2.29 (1.15–7.32)	−1.322/ 0.186		/	/	/	/	/	/
**TNF-α (pg/ml)**	8.19 (6.23–8.64)	NA	NA	9.42 (6.55–14.05)	6.86 (4.35–16.39)	−1.178/ 0.239	8.29 (7.60–13.29)	12.51 (6.02–12.20)	−0.138/ 0.890		/	/	/	/	/	/
**TGF-β1 (pg/ml)**	5,114 (2,496.50–5,751.50)	10,817.50 (10,445–10,917)	−0.712/ 0.476	1,319.69 (268.60–3,611)	152.73 (99.22–3,790.50)	1.517/ 0.129	2,032 (104.97–2,548)	1,514 (112.50–5,959)		−0.276/ 0.783	/	/	/	/	/	/
**TGF-β2 (pg/ml)**	412.21 (210.48–412.21)	NA	NA	/	/	/	/	/		/	/	/	/	/	/	/
**TGF-β3 (pg/ml)**	200.31 (147.58–209.12)	NA	NA	/	/	/	/	/		/	/	/	/	/	/	/

biochemistry and virus indicators: p < 0.010 was statistically significant; cytokines: p < 0.013 was statistically significant.

### Baseline predictors of ALT normalization at week 48 of ETV treatment

Univariate analysis showed that baseline HBsAg, HBeAg, and IL-10 were significantly correlated with ALT normalization at 48 weeks after ETV treatment (HBsAg OR = 4.058, 95% CI 1.080–15.245, *p* = 0.038; HBeAg OR = 1.002, 95% CI 1.000–1.004, *p* = 0.011; IL-10 OR = 0.985, 95% CI 0.973–0.997, *p* = 0.015). Multivariate analysis showed that baseline HBV DNA, HBeAg, and IL-10 levels were independent factors significantly related to whether ALT was normal or not after 48 weeks of ETV treatment (HBeAg OR = 1.003, 95% CI 1.001–1.006, *p* = 0.007; HBV DNA OR = 0.184, 95% CI 0.046–0.739, *p* = 0.017; IL-10 OR = 0.040, 95% CI 0.972–0.999, *p* = 0.040) (**Table 4**).

**Table 4:** Logistic regression analysis of the baseline predictors of ALT normalization at week 48 of ETV treatment.

Univariate analysis	OR	95% CI	*p*-value
**HBsAg (log10 IU/ml)**	4.058	1.080–15.245	0.038
**HBeAg (S/CO)**	1.002	1.000–1.004	0.011
**HBV DNA (log10 IU/ml)**	0.846	0.390–1.834	0.672
**ALT (U/L)**	0.999	0.997–1.001	0.238
**AST (U/L)**	0.997	0.995–1.000	0.076
**TBil (µmol/L)**	0.943	0.883–1.008	0.084
**ALB (g/L)**	1.080	0.953–1.224	0.229
**Flt-3L (pg/ml)**	0.984	0.939–1.040	0.559
**IFN-α2 (pg/ml)**	1.006	0.994–1.018	0.340
**IFN-γ (pg/ml)**	1.000	0.996–1.005	0.871
**IL-10 (pg/ml)**	0.985	0.973–0.997	0.015
**IL-17A (pg/ml)**	1.005	0.998–1.022	0.548
**IL-6 (pg/ml)**	1.061	0.930–1.211	0.380
**TNF-α (pg/ml)**	1.011	0.934–1.094	0.791
**TGF-β1 (pg/ml)**	1.000	1.000	0.443
**TGF-β2 (pg/ml)**	0.995	0.985–1.006	0.364
**TGF-β3 (pg/ml)**	1.011	0.955–1.069	0.713
**Multiple factor analysis**	OR	95% CI	*p*-value
**HBV DNA (log10 IU/ml)**	0.184	0.046–0.739	0.017
**HBeAg (S/CO)**	1.003	1.001–1.006	0.007
**IL-10 (pg/ml)**	0.040	0.972–0.999	0.040

cytokines: p < 0.013 was statistically significant.

## Discussion

Controlling HBV replication and improving body immunity are important means to reduce liver inflammation and prevent disease progression. Nucleoside (acid) analogues and interferon have become the first-line antiviral drugs for CHB (2, 6, 7). The target site of nucleoside (acid) analogues is the reverse transcriptase required in the process of HBV DNA replication (21). Nucleoside (acid) analogue therapy is fast-acting, safe, and well tolerated, but they are ineffective for covalently closed circular DNA (cccDNA) in the nucleus, and the viral load will rebound after drug withdrawal. Therefore, patients need to receive long-term drug treatment in most cases.

In this study, we chose high gene barrier resistance and potent ETV treatment, and the overall virological response was good. In our study, 87 of the 100 patients obtained a complete virological response. Although there was no significant difference between the groups, the baseline HBsAg, HBeAg, HBV DNA load, IL-10, and TGF-β1 levels in the complete viral response group were lower, and the level of ALT was higher than those in the incomplete viral response group.

High HBV DNA load, and high HBeAg and HBsAg levels may inhibit the immune cell function of patients, leading to the reduction of Flt-3L, IFN-γ, IL-17A, and other cytokines with virus clearance effect (8, 13, 22–24), and to the increase in the level of the most important cytokine for immunosuppression (IL-10) and other cytokines with immunosuppressive effect such as TGF-β1 (8, 13, 25–27). In this study, compared with the incomplete virus response group, the complete virus response group had lower HBV DNA load, lower HBeAg and HBsAg levels, lower IL-10 level, and lower TGF-β level, which indicate that patients with complete virus response may have better immune clearance ability against virus. HBeAg and IL-10 levels in the complete virological response group were lower than those in the incomplete response group at 3 months of treatment, while IFN-γ and IL-17A were higher. At 6 months of treatment, HBeAg in the complete virological response group was significantly lower than that in the incomplete virological response group, while IFN-α2, IFN-γ, and IL-17A were higher.

HBsAg and HBeAg levels at 9 and 12 months in the complete virological response group were significantly lower than those in the incomplete response group. The result suggests that the acquisition of complete virological response may be related to the decrease of antigen, the improvement of immune capacity (the increase of the cytokines with immunostimulatory effect), and the decrease of the cytokines with immunosuppressive effect (8, 13, 14, 25, 28, 29). However, it is difficult to clarify the causal relationship between the complete viral response and the increase in the level of the cytokines with immunostimulatory effect and the decrease in the level of the cytokines with immunosuppressive effect. Through univariate and multivariate analysis, no factors related to the virological response at 48 weeks were found, which may be related to the fact that ETV is a potent drug that strongly inhibits HBV replication, thus masking the impact of other factors on the antiviral effect.

The aim of antiviral therapy is to prevent or reduce the inflammation of liver tissue, and to block or delay the progress of liver disease by inhibiting the replication of virus and improving the immune control ability of virus (2, 6, 7). In our study, 90 of 100 patients (90%) achieved ALT normalization after 48 weeks of ETV treatment. The baseline HBsAg and HBeAg levels in the ALT normalization group were significantly lower than that in the non-normalization group. The baseline IL-10 and TGF-β1 in the ALT normalization group were lower than that in the ALT non-normalization group, while the baseline ALT level and IFN-α2 level were significantly higher. Compared with the ALT non-normalization group, the ALT normalization group had lower baseline HBsAg, HBeAg, IL-10, and TGF-β levels and higher baseline ALT level. This indicates that the ALT normalization group may have better baseline immune control ability (14, 15, 28), and the efficacy of NA treatment is related to the immune and viral factors of patients (2, 6, 7). At 3 and 6 months after treatment, the IFN-γ level of the ALT normalization group was significantly higher than that in the non-normalization group. Given that IFN-γ is an important cytokine for cellular immunity (14, 25, 28, 29), the increase in IFN-γ suggests that ETV can restore part of cellular immune function by reducing HBV DNA load (29).

Logistic regression analyzed risk factors related to whether the ALT is normal after 48 weeks of ETV treatment. Univariate analysis showed that low baseline levels of HBsAg, HBeAg, and IL-10 were significantly related to normal ALT at 48 weeks. Multivariate analysis showed that baseline HBV DNA, HBeAg, and IL-10 were significantly correlated with normal ALT at 48 weeks. After 48 weeks of treatment with ETV, normal ALT means good biochemical response and better control of hepatocyte necrosis and liver tissue inflammation. The results show that the effect of ETV treatment can be affected by baseline viral factors, the degree of liver tissue inflammation, and immune cytokines.

Although univariate analysis showed that baseline HBsAg, HBeAg, and IL-10 were heavily correlated with ALT normalization at 48 weeks after ETV treatment, the levels of HBeAg and HBsAg in the ALT normalization group were higher than those in ALT elevated group after 48-week ETV treatment, and the reason was still considered to be related to the immune function of patients. In patients with chronic HBV infection, the existence of liver diseases and progression are influenced by multiple factors. In terms of HBV, HBV replication can activate the response of host immune cells and cause liver cell necrosis and inflammation. On the other hand, HBV and its antigens can inhibit the immune cell function through multiple ways, making it difficult to clear the virus (14–17). Therefore, the decrease of HBV DNA level in most patients during NA treatment can reduce the stimulation of immune cells and the immune damage of hepatocytes, and thus the clinical manifestations are ALT normalization, and no decrease or even increase in HBsAg and HBeAg. However, in some patients, due to the decrease or even negative transformation of HBV level during NA treatment, the inhibition of immune cell function by HBV is alleviated, and the function of immune cells to inhibit viral replication and clear virus-infected cells is restored. The clinical manifestations are increased ALT, accompanied by decreased HBeAg and HBsAg levels. Thus, the results of this study showed that patients with elevated ALT during ETV treatment had lower HBeAg and HBsAg levels than those with normal ALT.

In conclusion, this prospective study shows that cytokine levels change dynamically in ETV antiviral therapy. Low baseline HBV DNA, HBeAg, and IL-10 levels were significantly associated with ALT normalization after 48 weeks of ETV treatment. The findings warrant further investigation with a large sample size in the future. In this study, we only investigated the kinetic changes of serum, virological, and immunological markers during ETV antiviral therapy in HBeAg-positive CHB patients. In the future, we expect similar studies to be conducted in HBeAg-negative CHB patients.

## Data availability statement

The raw data supporting the conclusions of this article will be made available by the authors, without undue reservation.

## Ethics statement

This study was reviewed and approved by The Ethics Committee of Beijing Ditan Hospital affiliated to Capital University of Medical Sciences. The patients/participants provided their written informed consent to participate in this study.

## Author contributions

ML, RS, YJ, WY and YX contributed to study concept and design. YG, LY, YanL, WD, TJ, XB, YaoL, LZ,GS, RL, SW, MC, MX and LH performed the data collection. ML, RS, YJ, WY and YX conducted data analysis. ML, YG, LY and YanL wrote the first draft. ML edited the English version. RS, YJ, WY and YX approved the submitted version after modification. All authors contributed to the article and approved the submitted version.

## Funding

This work was funded by the High-level Public Health Technical Personnel Training Program of Beijing Municipal Health Commission (2022-3-050), the Capital Health Research and Development of Special (2022-1-2172), the Digestive Medical Coordinated Development Center of Beijing Hospitals Authority (XXZ0302 and XXT28), Beijing Hospitals Authority Clinical Medicine Development of Special Funding Support (XMLX 202127), Project supported by Beijing Science and Technology Commission (Z211100002921059), and the National Science and Technology Major Project of China (2017ZX10201201-001-006, 2017ZX10201201-002-006, and 2018ZX10715-005-003-005).

## Conflict of interest

The authors declare that the research was conducted in the absence of any commercial or financial relationships that could be construed as a potential conflict of interest.

The editor declared a shared parent affiliation with the authors at the time of review.

## Publisher’s note

All claims expressed in this article are solely those of the authors and do not necessarily represent those of their affiliated organizations, or those of the publisher, the editors and the reviewers. Any product that may be evaluated in this article, or claim that may be made by its manufacturer, is not guaranteed or endorsed by the publisher.

## References

[1] Wang FS Fan JG Zhang Z Gao B Wang HY. The global burden of liver disease: the major impact of China. Hepatology (2014) 60:2099–108. doi: 10.1002/hep.27406 PMC4867229 25164003

[2] Wang GQ Wang FS Li TS. Guidelines for the prevention and treatment of chronic hepatitis b (version 2019). Chin J Hepatol (2020) 23:9–32. doi: 10.3969/j.issn.1672-5069.2020.01.044

[3] Croagh CM Lubel JS. Natural history of chronic hepatitis b: phases in a complex relationship. World J Gastroenterol (2014) 20:10395–404. doi: 10.3748/wjg.v20.i30.10395 PMC4130846 25132755

[4] Busch K Thimme R. Natural history of chronic hepatitis b virus infection. Med Microbiol Immunol (2015) 204:5–10. doi: 10.1007/s00430-014-0369-7 25540037

[5] Wu S Yi W Gao Y Deng W Bi X Lin Y. Immune mechanisms underlying hepatitis b surface antigen seroclearance in chronic hepatitis b patients with viral coinfection. Front Immunol (2022) 13:893512. doi: 10.3389/fimmu.2022.893512 35634301 PMC9130599

[6] European Association for the Study of the Liver Electronic address: easloffice@easloffice.eu European Association for the Study of the Liver. EASL 2017 clinical practice guidelines on the management of hepatitis b virus infection. J Hepatol (2017) 67:370–98. doi: 10.1016/j.jhep.2017.03.021 28427875

[7] Heimbach JK Kulik LM Finn RS Sirlin CB Abecassis MM Roberts LR. AASLD guidelines for the treatment of hepatocellular carcinoma. Hepatology (2018) 67:358–80. doi: 10.1002/hep.29086 28130846

[8] Li MH Zhang D Zhang L Qu XJ Lu Y Shen G. Ratios of T-helper 2 cells to T-helper 1 cells and cytokine levels in. Chin Med J (2017) 130:1810–5. doi: 10.4103/0366-6999.211541 PMC5547833 28748854

[9] Li MH Zhang L Zhang D Cao WH Qi TL Hao HX. Plasmacytoid dendritic cell function and cytokine network profiles in patients with acute or chronic hepatitis b virus infection. Chin Med J (2018) 131:43–9. doi: 10.4103/0366-6999.221275 PMC5754957 29271379

[10] Cao WH Li MH Pan CQ Lu Y Zhang L Ran CP. Quantitation of plasmacytoid dendritic cells in chronic hepatitis b patients with HBeAg positivity during PEG-IFN and entecavir therapy. J Interferon Cytokine Res (2018) 38:197–205. doi: 10.1089/jir.2018.0014 29791282

[11] Cao W Li M Zhang L Lu Y Wu S Shen G. The characteristics of natural killer cells in chronic hepatitis b patients who received PEGylated-interferon versus entecavir therapy. BioMed Res Int (2021) 2021:2178143. doi: 10.1155/2021/2178143 33575322 PMC7857883

[12] Cao W Xie S Zhang L Bi X Lin Y Yang L. Expression of functional molecule on plasmacytoid dendritic cells is associated with HBsAg loss in HBeAg-positive patients during PEG-IFN α-2a treatment. Front Immunol (2022) 13:891424. doi: 10.3389/fimmu.2022.891424 35663955 PMC9160736

[13] Li M Zhang L Xie S Sun F Zeng Z Deng W. Dynamic changes of cytokine profiles and virological markers associated with HBsAg loss during peginterferon alpha-2a treatment in HBeAg-positive chronic hepatitis b patients. Front Immunol (2022) 13:892031. doi: 10.3389/fimmu.2022.892031 35603222 PMC9114800

[14] Li MH Lu Y Zhang L Wang XY Ran CP Hao HX. Association of cytokines with alanine aminotransferase, hepatitis b virus surface antigen and hepatitis b envelope antigen levels in. Chin Med J (2018) 131:1813–8. doi: 10.4103/0366-6999.237394 PMC6071474 30058578

[15] Li MH Lu HH Chen QQ Lin YJ Zeng Z Lu Y. Changes in the cytokine profiles of patients with chronic hepatitis b during antiviral therapy. Biomed Environ Sci (2021) 34:443–53. doi: 10.3967/bes2021.061 34284852

[16] Guimond M Freud AG Mao HC Yu J Blaser BW Leong JW. *In vivo* role of Flt3 ligand and dendritic cells in NK cell homeostasis. J Immunol (2010) 184:2769–75. doi: 10.4049/jimmunol.0900685 PMC2924750 20142363

[17] Waskow C Liu K Darrasse-Jèze G Guermonprez P Ginhoux F Merad M. The receptor tyrosine kinase Flt3 is required for dendritic cell development in peripheral lymphoid tissues. Nat Immunol (2008) 9:676–83. doi: 10.1038/ni.1615 PMC2746085 18469816

[18] European Association for the Study of the Liver Asociacion Latinoamericana para el Estudio del Higado. EASL-ALEH clinical practice guidelines: Non-invasive tests for evaluation of liver disease severity and prognosis. J Hepatol (2015) 63:237–64. doi: 10.1016/j.jhep.2015.04.006 25911335

[19] Terrault NA Bzowej NH Chang KM Hwang JP Jonas MM Murad MH. AASLD guidelines for treatment of chronic hepatitis b. Hepatology (2016) 63:261–83. doi: 10.1002/hep.28156 PMC5987259 26566064

[20] Sarin SK Kumar M Lau GK Abbas Z Chan HL Chen CJ. Asian-Pacific clinical practice guidelines on the management of hepatitis b: a 2015 update. Hepatol Int (2016) 10:1–98. doi: 10.1007/s12072-015-9675-4 PMC4722087 26563120

[21] Pierra Rouviere C Dousson CB Tavis JE. HBV replication inhibitors. Antiviral Res (2020) 179:104815. doi: 10.1016/j.antiviral.2020.104815 32380149 PMC7293572

[22] Ma A Motyka B Gutfreund K Shi YE George R. A dendritic cell receptor-targeted chimeric immunotherapeutic protein (C-HBV) for the treatment of chronic hepatitis b. Hum Vaccin Immunother (2020) 16:756–78. doi: 10.1080/21645515.2019.1689080 PMC7227630 31687879

[23] De Pasquale C Campana S Barberi C Sidoti Migliore G Oliveri D Lanza M. Human hepatitis b virus negatively impacts the protective immune crosstalk between natural killer and dendritic cells. Hepatology (2021) 74:550–65. doi: 10.1002/hep.31725 PMC8295401 33482027

[24] Sahu U Biswas D Prajapati VK Singh AK Samant M Khare P. Interleukin-17-A multifaceted cytokine in viral infections. J Cell Physiol (2021) 236:8000–19. doi: 10.1002/jcp.30471 PMC8426678 34133758

[25] Li MH Lu Y Sun FF Chen QQ Zhang L Lu HH. Transforming growth factor β as a possible independent factor in chronic hepatitis b. Arch Virol (2021) 166:1853–8. doi: 10.1007/s00705-021-05062-6 33871695

[26] Martinez-Espinosa I Serrato JA Ortiz-Quintero B. Role of IL-10-Producing natural killer cells in the regulatory mechanisms of inflammation during systemic infection. Biomolecules (2021) 12 :4. doi: 10.3390/biom12010004 35053151 PMC8773486

[27] Li Y Fan W Link F Wang S Dooley S. Transforming growth factor β latency: A mechanism of cytokine storage and signalling regulation in liver homeostasis and disease. JHEP Rep (2022) 4:100397. doi: 10.1016/j.jhepr.2021.100397 35059619 PMC8760520

[28] Li MH Chen QQ Zhang L Lu HH Sun FF Zeng Z. Association of cytokines with hepatitis b virus and its antigen. J Med Virol (2020) doi: 10.1002/jmv.26301 32662892

[29] Shiue SJ Cheng CL Shiue HS Chen CN Cheng SW Wu LW. Arthrospira enhances seroclearance in patients with chronic hepatitis b receiving nucleos(t)ide analogue through modulation of TNF-α/IFN-γ profile. Nutrients (2022) 14 :2790. doi: 10.3390/nu14142790 35889747 PMC9325115

